# CNS Vasculitis Associated with Waldenström Macroglobulinemia

**DOI:** 10.1155/2016/2510573

**Published:** 2016-10-12

**Authors:** Tanawan Riangwiwat, Chris Y. Wu, Alberto S. Santos-Ocampo, Randal J. Liu, Aaron M. McMurtray, Beau K. Nakamoto

**Affiliations:** ^1^Department of Medicine, University of Hawaii, Honolulu, HI, USA; ^2^Department of Rheumatology, Straub Clinics and Hospital, Honolulu, HI, USA; ^3^Department of Oncology, Straub Clinics and Hospital, Honolulu, HI, USA; ^4^Department of Neurology, Harbor-UCLA Medical Center, Torrance, CA, USA; ^5^Department of Neurology, Straub Clinics and Hospital, Honolulu, HI, USA

## Abstract

Waldenström macroglobulinemia (WM) is an indolent B cell lymphoproliferative disorder with monoclonal IgM secretion. We present a patient with WM who presented with multifocal acute cortical ischemic strokes and was found to have central nervous system (CNS) vasculitis. Workup was negative for cryoglobulins and hyperviscosity syndrome. Immunosuppression with intravenous steroids and cyclophosphamide stabilized the patient's mental status and neurologic deficits. On followup over 7 years, patient gained independence from walking aids and experienced no recurrences of CNS vasculitis. To our knowledge, CNS vasculitis in a WM patient, in the absence of cryoglobulins, has not been reported. Immunosuppression is the preferred treatment.

## 1. Introduction

Waldenström macroglobulinemia (WM) is an indolent B cell lymphoproliferative disorder associated with hypersecretion of serum monoclonal IgM protein [1]. Central nervous system (CNS) vasculitis comprises a broad group of diseases that lead to inflammation and destruction of blood vessels in the brain, spinal cord, or meninges [2]. We present a case of a patient with WM who developed CNS vasculitis.

## 2. Case Report

A 66-year-old woman with WM, hypertension, hyperlipidemia, and chronic anemia was referred by her primary care provider for evaluation of acute altered mental status. Symptomatology additionally included right-left disorientation, difficulty concentrating, blurry vision, and poor coordination (described as difficulty grabbing objects). Six years prior to presentation, she was found to be anemic [hemoglobin 9.1 g/dL, reference range 11.5–15.5] on routine medical evaluation. White blood cell and platelet counts were within normal limits. Further evaluation demonstrated an IgM kappa monoclonal protein. IgM was 659 mg/dL [reference range 57–341]. IgG was 448 mg/dL [reference range 780–1656]. IgA, calcium, creatinine, serum viscosity, and beta-2-microglobulin were within normal limits. There were no osteolytic lesions on skeletal bone survey. Bone marrow biopsy revealed infiltration by lymphoplasmacytoid cells. Flow cytometric immunophenotyping revealed that the cells were positive for CD19, CD20 and negative for CD5, CD10, and CD23. She was initially treated with rituximab and then maintained on thalidomide until 2 months prior to presentation. In the month prior to presentation, she suffered spontaneous atraumatic fractures of the left upper sacrum and right scapular presumed to be pathologic fractures secondary to WM, for which she was started on narcotic pain medications and later hospitalized with acute kidney injury from nonsteroidal anti-inflammatory drug usage. Current medications included aspirin, estrogen patch, famotidine, calcitonin, zoledronic acid, iron sulfate, omega-3 fatty acids, antihypertensive medications, and a variety of oral/transdermal narcotic medications. Family history was significant for mother with multiple myeloma. Patient was a practicing attorney at the time of presentation.

Vital signs were within normal limits. On physical exam, patient was oriented to person and place only. She was unable to spell the word “WORLD” backwards, though she had normal repetition and no right-to-left confusion. She had difficulty maintaining concentration to comply with exam, and thus, visual fields were not assessed. Optic ataxia was present. Cranial nerves III–XII were intact. Ophthalmologic evaluation did not reveal uveitis, retinal hemorrhages, or optic nerve head edema. Patient had normal strength, sensation, cerebellar tests, and Babinski reflex. She had symmetric 3+ reflexes at biceps and patellar. Hoffman reflex was present bilaterally, and gait was antalgic.

**Figure 1:**
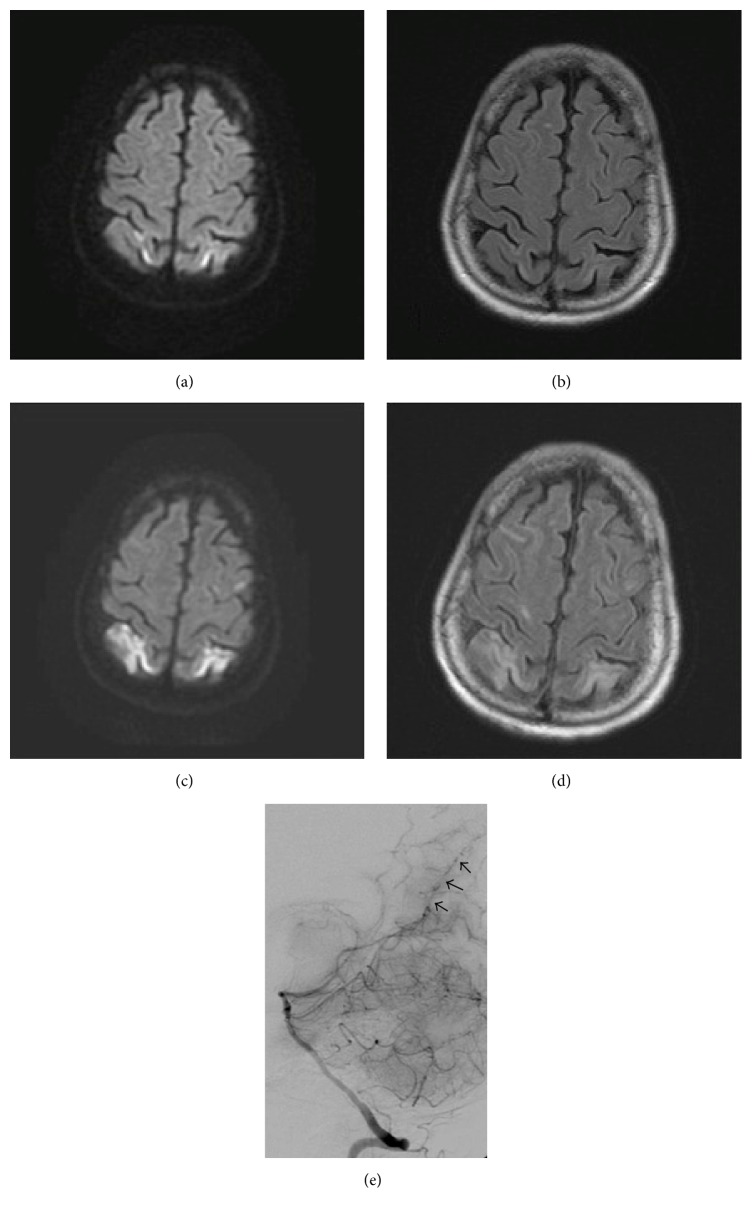
Relevant brain and blood vessel imaging highlighting vasculitic cause of multifocal strokes. MRI on day of admission with diffusion scan (a) significant for curvilinear increased signal in the cortical surface of both anterior parietal lobes extending into the postcentral gyrus on left, with additional foci of restricted diffusion within the periventricular white matter in both hemispheres (left > right). Subtle signal abnormalities are seen along cortical surface primarily in both anterior parietal lobes on FLAIR imaging (b). MRI performed 2 days after initial imaging showed progression of FLAIR signal (d) abnormalities with reduced diffusion (c) consistent with multifocal cortical infarcts involving the posterior frontal, occipital, left temporal, left frontal, and deep bilateral white matter. Cerebral angiogram (e) showing multifocal distal small vessel moderate to severe vasculitis predominantly involving the posterior circulation. Arrows point to areas of vessel beading and irregularity.

Noncontrast head computed tomography (CT) was within normal limits. Basic labs were significant for anemia and mildly reduced glomerular filtration rate. Patient was admitted for presumed delirium secondary to polypharmacy from narcotic medications. Magnetic resonance imaging (MRI) of the brain obtained later on the day of admission showed acute nonenhancing symmetric infarctions involving the cortical surface of the anterior parietal lobes (Figures 1(a) and 1(b)). Magnetic resonance venography was normal. Lumbar puncture (LP) showed mildly elevated proteins, but no leukocytosis or abnormal cytology. WM labs were significant for elevated IgM of 2250 mg/dL [reference range 40–260], normal IgA level, and low IgG of 236 mg/dL [reference range 741–1861]. Serum viscosity was 1.89 centipoise [reference range of 1.10 to 1.80]. Bacterial and viral infectious workup as well as autoimmune and systemic vasculitis workup (including cryoglobulinemia) of cerebrospinal fluid (CSF) and serum was negative. Electroencephalogram showed diffuse slowing.

Due to development of acute left hemiparesis, repeat brain MRI was obtained two days after admission, which showed progression of FLAIR signal abnormalities with reduced diffusion consistent with multifocal cortical infarctions (Figures 1(c) and 1(d)). Magnetic resonance angiography showed small peripheral branches of the cerebral arteries, suspicious for vasculitis. Patient was empirically started on intravenous steroids for CNS vasculitis. Cerebral angiogram performed the next day confirmed multifocal small vessel vasculitis predominantly involving the posterior circulation (Figure 1(e)). Cyclophosphamide was added to the patient's steroid regimen, and the patient's mental status and neurologic deficits gradually improved, though she had residual left hemiparesis. Repeat MRI showed evolution and stabilization, respectively, of ischemic infarcts. Patient was discharged to rehabilitation center for physical therapy with oral prednisone and cyclophosphamide, both of which were successfully tapered/discontinued in the year after discharge.

On followup over 7 years, patient developed peripheral distal axonal sensorimotor neuropathy that was treated with pregabalin and eventually gained independence from walker/canes with ambulation. Although the patient initially had visual complaints, her peripheral vision improved gradually during followup. She experienced no recurrences or exacerbations of WM or CNS vasculitis. She continued to have an IgM kappa monoclonal protein. IgM was 560 mg/dL [reference range 40–260], IgG was 271 mg/dL [reference range 741–1861 mg/dL], and IgA was 51 mg/dL [reference range 70–463]. White blood cell count was 13 × 10^9^/L [reference range 3.8–11.2]. Hemoglobin, platelet count, calcium, creatinine, serum viscosity, and beta-2-microglobulin were within normal limits.

## 3. Discussion

Waldenström macroglobulinemia (WM) is an indolent B cell lymphoproliferative disorder characterized by a monoclonal IgM protein in the serum and more than 10% infiltration of the bone marrow by lymphoplasmacytic cells [1]. With an age-adjusted incidence rate of 3.4 per million person years in males and 1.7 in females, WM usually affects men above the age of 60 years [1]. Treatment is not initiated until patients become symptomatic, most commonly for anemia. Neurologic complications occur in up to half of patients, mostly manifesting as peripheral neuropathy or as clinical symptoms of hyperviscosity [1]. Clinically significant serum viscosity (typically > 4 centipoises) leads to vasoocclusive events with retinal, neurologic, and bleeding symptomatology [1]. Pathophysiologically, nervous system involvement can occur due to nonspecific IgM deposition, antibody-mediated damage to nerves or vessels, and direct infiltration of the neuraxis (Bing-Neel syndrome, < 50 cases described) [1].

Vasculitis has been associated with WM only in the presence of cryoglobulins and immunoglobulins that reversibly precipitate at temperatures < 37°C and that can lead to immune complex-mediated vasculitis [3]. Our patient, however, tested negative for cryoglobulins and, additionally, lacked symptoms suggestive of cryoglobulinemia such as Raynaud's phenomenon, purpura, arthralgia, acrocyanosis, or skin ulceration, making a false negative result highly unlikely [3]. Workup was also negative for secondary autoimmune, systemic vasculitic, and infectious causes of CNS vasculitis, and additionally, our patient's serum viscosity value was not elevated appreciatively. However, cutaneous and systemic vasculitis has been uncommonly associated with other lymphoproliferative disorders in the absence of cryoglobulins. For instance, cases of hairy cell leukemia associated with polyarteritis nodosa have been reported, and that of Hodgkin's disease and granulomatous angiitis of the CNS as well [4]. Therefore, it is plausible that WM may lead to CNS vasculitis independent of cryoglobulins.

The mechanism of CNS vasculitis in our case remains speculative, and in fact, the exact mechanism of nervous system damage in general in patients with WM remains speculative [1]. Given that WM is known to cause autoimmune phenomena, potential causes include antibody-mediated damage (to a potential CNS blood vessel antigen), antibody-antigen deposits, cell-mediated damage from perivascular infiltration, and monoclonal protein deposition in CNS blood vessels with elicitation of inflammation and occlusion [1, 4]. In our case, there are also no clear identifiable triggers. Our patient had been on thalidomide 2 months before presentation, and there are cases of association between thalidomide and leukocytoclastic vasculitis in patients with lymphoproliferative disorders [5]. However, cryoglobulinemia testing was not reported in the cited cases, and contradictory case reports also exist [5]. It appears more likely that the termination of maintenance immunosuppressive therapy in our patient led to the development of CNS vasculitis as vasculitis is an autoimmune phenomenon. Importantly, our patient experienced stabilization and improvement of symptoms upon treatment with steroids and cyclophosphamide, both of which are also useful in managing WM. She has not experienced a recurrence of CNS vasculitis symptoms, and she continues to be closely followed up for recurrence of WM.

In summary, we present a patient with CNS vasculitis associated with Waldenström macroglobulinemia. Further studies examining the mechanism of vasculitis and central nervous system pathophysiology in Waldenström macroglobulinemia are needed.
